# Impact of surface structure and feed gas composition on *Bacillus subtilis* endospore inactivation during direct plasma treatment

**DOI:** 10.3389/fmicb.2015.00774

**Published:** 2015-08-06

**Authors:** Christian Hertwig, Veronika Steins, Kai Reineke, Antje Rademacher, Michael Klocke, Cornelia Rauh, Oliver Schlüter

**Affiliations:** ^1^Leibniz Institute for Agricultural EngineeringPotsdam-Bornim, Germany; ^2^Department of Food Biotechnology and Food Process Engineering, Berlin University of TechnologyBerlin, Germany

**Keywords:** cold plasma, spore inactivation, inactivation mechanism, DNA damage, qPCR

## Abstract

This study investigated the inactivation efficiency of cold atmospheric pressure plasma treatment on *Bacillus subtilis* endospores dependent on the used feed gas composition and on the surface, the endospores were attached on. Glass petri-dishes, glass beads, and peppercorns were inoculated with the same endospore density and treated with a radio frequency plasma jet. Generated reactive species were detected using optical emission spectroscopy. A quantitative polymerase chain reaction (qPCR) based ratio detection system was established to monitor the DNA damage during the plasma treatment. Argon + 0.135% vol. oxygen + 0.2% vol. nitrogen as feed gas emitted the highest amounts of UV-C photons and considerable amount of reactive oxygen and nitrogen species. Plasma generated with argon + 0.135% vol. oxygen was characterized by the highest emission of reactive oxygen species (ROS), whereas the UV-C emission was negligible. The use of pure argon showed a negligible emission of UV photons and atomic oxygen, however, the emission of vacuum (V)UV photons was assumed. Similar maximum inactivation results were achieved for the three feed gas compositions. The surface structure had a significant impact on the inactivation efficiency of the plasma treatment. The maximum inactivation achieved was between 2.4 and 2.8 log_10_ on glass petri-dishes and 3.9 to 4.6 log_10_ on glass beads. The treatment of peppercorns resulted in an inactivation lower than 1.0 log_10_. qPCR results showed a significant DNA damage for all gas compositions. Pure argon showed the highest results for the DNA damage ratio values, followed by argon + 0.135% vol. oxygen + 0.2% vol. nitrogen. In case of argon + 0.135% vol. oxygen the inactivation seems to be dominated by the action of ROS. These findings indicate the significant role of VUV and UV photons in the inactivation process of *B. subtilis* endospores.

## Introduction

In recent years, the application of cold atmospheric pressure plasma (CAPP) for the decontamination of food products, food packing material and/or food contact surfaces raised in attention (Pankaj et al., 2014; Schlüter and Fröhling, 2014). Plasma is in general an at least partially ionized gas, which contains charged particles such as ions and electrons as well as neutral species such as atoms, molecules, and radicals, furthermore also UV photons. Depending on their thermodynamic properties plasmas can be classified as thermal and non-thermal plasmas (Schlüter et al., 2013).

Thermal plasmas are characterized by a local thermodynamic equilibrium between the electrons, ions and neutral species, whereby the temperature of the plasma can reach several 1000 kelvins under atmospheric pressure (Moreau et al., 2008). In non-thermal plasma, there is a significant difference between the electron temperature and the temperature of the charged particles and bulk gas. The electron temperature can reach several 1000 kelvins, whereas the bulk gas temperature can be closed to ambient. These so called “cold” plasmas can be directly applied also to thermal sensitive surfaces (Ehlbeck et al., 2011).

Under atmospheric condition cold plasmas can be generated using different set-ups, such as dielectric barrier discharges or plasma jet systems (Surowsky et al., 2014). Various studies showed already the antimicrobial potential of different CAPP applications (Niemira, 2012), whereby the different reactive species inside the plasma, neutral and charges particles, UV photons and also irradiated heat, are responsible for the antimicrobial effect of the plasma application (Laroussi, 2002; Moisan et al., 2002). The obtained composition of the generated plasma depends on the plasma source, feed gas and also on operation conditions, e.g., energy input (Weltmann et al., 2008; Ehlbeck et al., 2011; Reineke et al., 2015).

The potential of several cold plasma applications to inactivate different endospores on various matrices was shown in other studies already (Lassen et al., 2005; Boudam et al., 2006; Deng et al., 2006; Brandenburg et al., 2009; Kim et al., 2014; Hertwig et al., 2015a,b; Reineke et al., 2015). Nevertheless, the mechanisms leading to the inactivation of endospores are not clear and are still under investigations. The inactivation behavior of endospores by plasma treatment is often described by biphasic inactivation kinetics (Moreau et al., 2000; Brandenburg et al., 2009; Reineke et al., 2015). These biphasic inactivation kinetics probably indicate the involvement of different inactivation effects of the cold plasma, like the inactivation by DNA damage due to emitted UV photons and the decomposition of microorganisms through photodesorption and etching. Whereas photodesorption is a UV-induced erosion of the cell, where UV photons break chemical bonds and lead to the formation of volatile compounds. Etching, however, is the adsorption of reactive species on microorganisms, leading to chemical reaction and the formation of volatile compounds (Moisan et al., 2002).

The main subject of the ongoing controversy regarding the CAPP based inactivation of microorganisms is the role of the generated UV and vacuum (V)UV photons. UV and VUV photons are known to induce strand breaks and other damages in DNA in the cell. Furthermore UV photons with wavelength below 275 nm can break C–C or C–H bonds (Brandenburg et al., 2009) and hence affect protein and other macromolecules structures and functioning. Most of the published studies claim that under atmospheric conditions UV photons play only a minor role in the inactivation process (Laroussi and Leipold, 2004; Perni et al., 2007; Lu et al., 2008; Knorr et al., 2011), since major quantities of (V)UV photons are only emitted in low-pressure or vacuum plasma systems. Nevertheless, some groups showed that UV photons can dominate the inactivation process (Boudam et al., 2006; Reineke et al., 2015).

The structure of the contaminated surface has also a certain impact on the spore inactivation efficiency of the CAPP treatment, as rough surfaces with pits and cracks can hinder the inactivation of microorganisms (Surowsky et al., 2014). In most cases endospores were inoculated and plasma treated on smooth surfaces such as glass, polyethylene strips, polycarbonate membranes, or polymer foils (Heise et al., 2004; Deng et al., 2006; Brandenburg et al., 2009; Reineke et al., 2015). However, studies investigating the inactivation of endospores on different surfaces are scarce.

In this study the effect of different structured surfaces (glass petri-dishes, glass beads and peppercorns) concerning the inactivation of *Bacillus subtillis* endospores during CAPP treatment using a radio frequency plasma jet was investigated and to ensure comparable results all samples were inoculated with a similar endospore density. The selection of different surfaces, from a simple even glass surface via a spherical model (glass beads) to a real food matrix (peppercorns), in combination with an inoculation of comparable spore density enable a closer insight into the surface-related inactivation effect of *B. subtilis* endospores. The samples were treated using three different feed gas compositions, in order to vary the composition of the generated plasma and also the focus between the different involved mechanisms in endospore inactivation. Considering that the DNA play likely a considerable role during the inactivation process by CAPP, the quantification of DNA damage during the plasma exposure may help to closer understand the plasma based mechanisms responsible for the inactivation of endospores. Therefore a quantitative polymerase chain reaction (qPCR) based ratio detection system was established, which detected the degree of *B. subtilis* DNA damage during the CAPP treatment.

## Materials and Methods

### *Bacillus subtilis* Endospore Preparation

The endospore forming *B. subtilis* strain PS832 was used in this study. *B. subtilis* was sporulated using the method previously published by Nicholson and Setlow (1990). Sporulation was induced on 2x SG medium agar plates at 37°C without addition of antibiotics. After sporulation, the endospores were harvested with distilled water. The obtained suspension was washed and cleaned with cold distilled water by repeated centrifugation (threefold at 5000 *g*) and intermittently treated with ultrasonic (1 min). The cleaned endospore suspension contained ≥95% phase bright endospores and nearly no endospore agglomerates. The endospores were stored in the dark at 4°C, until needed.

### Sample Preparation

In this study three different surfaces, such as glass petri-dishes (30 mm diameter), glass beads and whole black peppercorns (*Piper nigrum*), were used. Peppercorns were purchased from JJ Albaracin (Murcia, Spain). To ensure comparable results between the different surfaces, all samples were inoculated with an endospore density of about 4^∗^10^6^ endospores cm^-2^, which is comparable to the native microbial load of black peppercorns (Hertwig et al., 2015a). Therefore, the surface of the peppercorns was measured using a particle analyzer PartAn 3001L (AnaTec GmbH, Duisburg, Germany). 1 g of peppercorns had a surface of 18.4 cm^2^. The glass beads had a diameter of 5 mm. For the inoculation with *B. subtilis* endospores, 3.5 g of sterile peppercorns and 82 sterile glass beads, which had a similar surface of 64.4 cm^2^, were placed into a sterile beaker and 175 μL stock endospore suspension was added. The beaker was placed on an automatic stirrer and shaken for 4 min at 400 rpm to obtain a homogenous coating of the microorganisms on the samples surface. The inoculated samples were placed under a clean bench for drying at room temperature for 30 min. Regarding the inoculation of the glass petri-dishes the stock endospore suspension was diluted 1:5 with ACES-buffer (pH 7). An aliquot of 300 μL diluted endospore suspension was mixed with 700 μL ethanol (96%). 35 μL of the ethanolic endospore suspension were spread on an area of 1.5 cm^2^ of the glass petri-dishes.

### Plasma Source and Plasma Treatment

The radio frequency (rf) plasma jet equipment used in this study was described elsewhere in detail (Brandenburg et al., 2007). The apparatus consists of a ceramic nozzle (nozzle tip diameter ∼7 mm) with a needle electrode inside, a grounded ring electrode at the nozzle outlet, an rf-generator and a gas supply system. The rf-voltage is coupled with the needle electrode. The plasma is generated at the tip of this electrode and expands into the air outside the nozzle with a length of up to 15 mm. Prior to plasma treatment, the atmospheric pressure plasma jet was let run at experimental conditions for 15 min to allow preheating and passivation of the electrodes. Argon with additional mixing of 0.2% vol. nitrogen and/or 0.135% vol. oxygen was used as feed gas with a gas flow of 10 standard liter per minute and an operation power of 30 W. 1 g inoculated peppercorns and 23 inoculated glass beads were placed in individual sterile glass petri-dishes (30 mm diameter) and placed on an automatic stirrer below the collimated plasma beam with a distance of 12 mm to the nozzle outlet. The peppercorns were treated up to 15 min, glass beads up to 10 min, respectively. The inoculated glass petri-dishes were also plasma treated with a distance of 12 mm to the nozzle outlet, up to 5 min. A direct contact between the plasma and the surface of the glass petri-dishes was avoided, to prevent the endospores from abrasion by the plasma filaments. All treatments were done at least in quadruplicate.

### Optical Emission Spectroscopy

A Black Comet UV-VIS Spectrometer (StellarNet, Inc., Tampa, FL, USA) equipped with a F400 UV-VIS-SR fiber optic and a quartz lens was used to measure the emission spectrum of the direct CAPP set-up. The spectrum was measured in the range from 190 to 850 nm. The distance from the middle of the nozzle outlet to the middle of the lens was 10 mm in vertical and 12 mm in the horizontal axis. The spectrum was measured 10 times with an integration time of 100 ms. The average spectrum was base-line corrected and normalized (between λ = 450–470 nm) using a self-written LabVIEW routine.

### Viable Cell Counts

After the plasma treatment, the viable cell count was determined by standard cell culture methods. Therefore, the glass petri-dishes were filled with 1 ml ACES-buffer and four sterile glass beads were added. The *B. subtilis* endospores were resuspended by continuously shaking (250 rpm) for 30 min. The recovery of the endospores from the peppercorns and glass beads was carried out by shaking the samples in 4 ml ACES-buffer for 3 min at 400 rpm. The obtained suspensions were serially diluted in ACES-buffer and every dilution was plated on nutrient agar plates (Carl Roth GmbH, Karlsruhe, Germany) in duplicates. The plates were incubated at 37°C and the colony forming units (cfu) were counted after 24 and 48 h. The obtained inactivation kinetics were modeled with GInaFiT (Geeraerd and Van Impe Inactivation Model Fitting Tool), a freeware applet for Microsoft Excel, using a biphasic inactivation model (Cerf, 1977). In this model, the relation between the survival and exposure time is given by following equation:

$$\log_{10}S(t)=\varphi \cdot e^{k_{1}}\cdot {\;}^{t}+(1-\varphi )\cdot e^{k_{2}}\cdot {\;}^{t}$$

where S(t) is N(t)/N0, with N(t) as the number of colony forming units at the time t and N0 as the initial number of colony forming units. φ is the fraction of the initial population in a major population and (1-φ) is the fraction of the initial population in a minor population; k_1_ and k_2_ are the specific inactivation rates of the two populations.

### Infrared Temperature Imaging

During the plasma treatments, the surface temperature of the glass petri-dishes, glass beads and peppercorns was recorded by an infrared camera (ThermaCam 500, Flir, Frankfurt am Main, Germany) in triplicates. The emissivity of the glass petri-dishes and glass beads was set to 0.94 and 0.96 for peppercorns (BARTEC Messtechnik und Sensorik, 2001). The camera was installed from above at a distance of 1 m to the plasma treated sample; infrared images were taken at a frequency of 1 Hz. To exclude thermal inactivation effect, *B. subtilis* endospores inoculated on the three used material were thermal treated at the highest peak temperature measured during the plasma treatment, according to the corresponding plasma treatment time. The samples were placed in a heating and drying oven UT 20 (Heareus Instruments GmbH, Hanau, Germany) and the surface temperatures of the samples were measured using fiberglass-encased K-type thermocouples connected with a USB data acquisition system (Personal Daq/56, SynoTECH, Hückelhofen, Germany) and DASYLab 13.0 software.

### Determination of Endospore DNA Damage

A qPCR based ratio detection system (Bauer et al., 2004; Roth et al., 2010) was used to determine the degree of DNA damage of the *B. subtilis* endospores after plasma treatment. The recovered endospore suspensions were pooled and collected by centrifugation (10 min; 10,000 *g*; 4°C). In case of the glass petri-dishes recovered endospore suspension of four replicates were pooled together. For chemical decoating, the pellet was suspended in 200 μL of 50 mmol L^-1^ Tris-HCl (pH 8.0), which contains 8 mol L^-1^ urea, 1% sodium dodecyl sulfate, 10 mmol L^-1^ EDTA and 50 mmol L^-1^ dithiothreitol. After 90 min incubation at 37°C, the decoated endospores were washed three times by repeated centrifugation (10 min; 10,000 *g*; 4°C) with cold, sterile water (Fairheadt et al., 1993). By suspending the endospores in 200 μL STE-buffer (150 mmol L^-1^ NaCl, 10 mmol L^-1^ Tris-HCl, pH 8.0; 10 mmol L^-1^ EDTA) containing 2 mg mL^-1^ lysozyme and incubating at 37°C for 60 min, the disruption of the endospores was accomplished. From the disrupted endospores, chromosomal DNA was purified using the High Pure PCR Template Preparation Kit (Roche, Penzberg, Germany). The concentration of the DNA was determined using a NanoDrop 3300 fluorospectrometer (Thermo Fisher Scientific, Inc., Waltham, MA, USA) applying the PicoGreen^®^ dsDNA assay (Life Technologies GmbH, Darmstadt, Germany). The method used for the DNA damage determination was established by Roth et al. (2010) and optimized for the qPCR system used in this study. It can be assumed, that the plasma treatment causes randomly distributed defects along the DNA double strands, accordingly increases the probability of the detecting such defects with increasing length of the examined DNA fragment. Two PCR primer pairs Bs_dnaK855f (5′-CACAATGGGTCCTGTCCGTC-3′)/Bs_dnaK1254r (5′-AGACATTGGGCGCTCACCT-3′) and Bs_dnaK1154f (5′-ACACGACGATCCCAACAAGC-3′)/Bs_dnaK1254r were used to amplify a 400 bp reporter and an internal 101 bp fragment from the *dna*K locus. The 101 bp fragment was used as an internal standard. Both fragments were amplified on a CFX96 Touch^TM^ real-time PCR detection system (Bio-Rad Laboratories GmbH, München, Germany) in separate 20 μL volume reactions, each in triplicates. Per reaction 10 μL SYBR Green reagent (Quiagen, Hilden, Germany), 0.2 μmol L^-1^ of each primer and 0.10–0.15 ng templates DNA was used.

A 10-fold dilution series of a *B. subtilis* PS832 plasmid including the target fragment were used as an external standard and to determine the absolute copy number. The plasma treatment may cause cross-links between the DNA and other endospore components, thus influencing the quality and efficiency of DNA extraction and also unpredictably affecting the detection of the target fragments by PCR (Roth et al., 2010). The applied qPCR based ratio detection system (Roth et al., 2010) take this into account, whereby the target fragment copy numbers cn_400_ were normalized to those of the internal 101 bp fragment cn_101_. Thus, the degree of DNA damage was expressed as a ratio (CN) between the detected 400 and 101 bp fragment copy numbers. For non-degraded DNA is the resulting ratio equal to 1 and decreases with increasing degrees of DNA damage. This ratio detection system can only be used for qualitative evaluation, because the correlation between actual degree of DNA damage and the ratio is unknown. This method allows the detection of various DNA damages like double or single strand breaks and thymidine dimers (Roth et al., 2010).

## Results

### Surface Temperature during CAPP Treatment

The average surface temperatures measured during the CAPP treatment are shown in **Table 1**. The CAPP treatment of glass beads leads to maximum local temperatures up to 90.1°C, the ones for the glass petri-dishes and peppercorns were slightly lower. The average surface temperature measured directly after the treatment was in the range of 56.9–75.2°C. Considering the measured surface temperatures, this CAPP application cannot be classified as a non-thermal treatment. To exclude thermal inactivation effects *B. subtilis* endospores inoculated on the three different sample types were thermal treated at 90°C in a heat and drying oven, according to the maximum CAPP treatment time. The thermal treatment resulted in no considerable inactivation; only for glass beads an inactivation of 0.2 log_10_ was obtained. However, the temperatures during the CAPP treatment may support the inactivation; nevertheless this effect should be comparable on the different treated surfaces due to the similar maximum local temperatures.

**Table 1:** Mean surface temperatures (±SD) before and after CAPP treatment.

	Starting temperature [°C]	Temperature after CAPP treatment [°C]	Maximum temperature during CAPP treatment [°C]
Glass petri-dishes	29.8 (±3.5)	56.9 (±1.0)	82.3 (±2.3)
Glass beads	27.7 (±0.4)	75.2 (±1.3)	90.1 (±0.2)
Peppercorns	28.3 (±1.7)	63.8 (±4.2)	88.3 (±0.6)

### Characterization of Reactive Plasma Species

**FIGURE 1:**
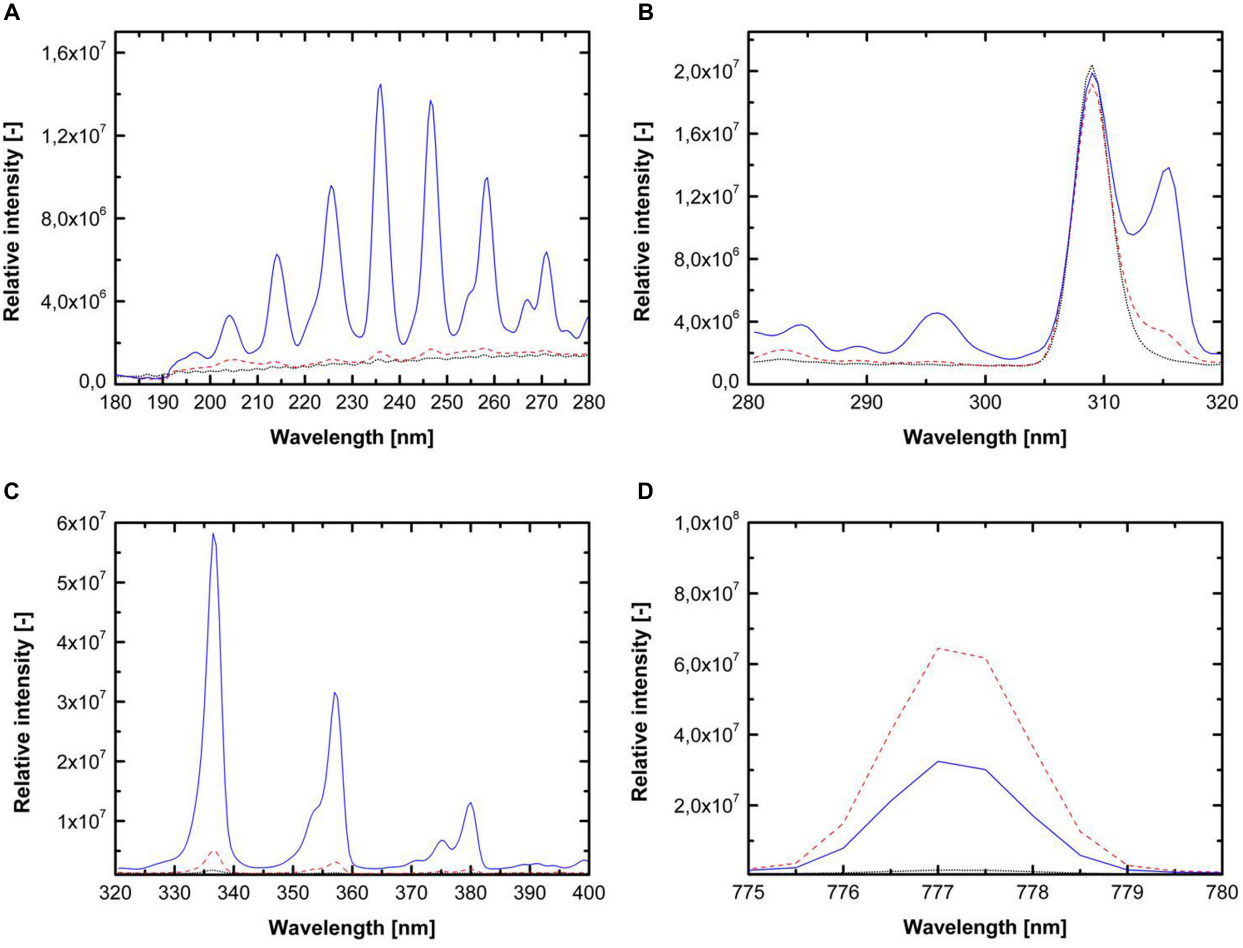
**Emission spectra for pure argon (dotted black line), argon + 0.135% vol. oxygen (dashed red line) and argon + 0.135% vol. oxygen + 0.2% vol. nitrogen (solid blue line) for wavelength from **(A)** 180–280 nm (UV-C light), **(B)** 280–320 nm (UV-B light), **(C)** 320–400 nm (UV-A light), and **(D)** 775–780 nm (atomic oxygen emission)**.

Three different feed gas compositions (1. pure argon, 2. argon + 0.135% vol. oxygen and 3. argon + 0.135% vol. oxygen + 0.2% vol. nitrogen) were used for detailed investigation of the involved inactivation mechanisms. Reineke et al. (2015) systematically investigated the emission intensity of argon plasma with the admixture of different oxygen and nitrogen concentration and showed that plasma running with argon + 0.135% vol. oxygen emitted a high amount of reactive oxygen species (ROS), whereas plasma running with argon + 0.135% vol. oxygen + 0.2% vol. nitrogen was characterized by the highest emission of UV-C photons. The emission spectra of the used plasmas, generated with the chosen gas compositions, are shown in **Figure 1**. The addition of oxygen and nitrogen causes significant changes in the emission spectra. In case of pure argon, molecular bands of oxygen, nitrogen, and other species were also detected due to interactions of the argon plasma with the surrounding air. The use of argon + 0.135% vol. oxygen + 0.2% vol. nitrogen resulted in considerable emission in the UV-C range (**Figure 1A**), whereas the emission of UV-C photons was negligible for the two other feed gas compositions. **Figure 1B** shows the emission intensity of the UV-B range, which was dominated by the signal of OH radicals with the maximum of 309 nm. The addition of oxygen and nitrogen resulted in no significant changes in the emission intensity of OH radicals. The emission spectrum from 320 to 400 nm (UV-A, **Figure 1C**) is dominated by molecular bands of the second positive system of N_2_. The emissions for pure argon and argon + 0.135% vol. oxygen were negligible comparing to the use of argon + 0.135% vol. oxygen + 0.2% vol. nitrogen. **Figure 1D** shows the emission intensity from 775 to 780 nm, this wavelength range is characterized by the atomic oxygen band at 777 nm, and depicts considerable variations depending on the gas composition. The feed gas composition argon + 0.135% vol. oxygen emitted the highest photon intensity.

### Effect of Feed Gas Composition and Surface Structure on the Inactivation of *B. subtilis* Endospores

**FIGURE 2:**
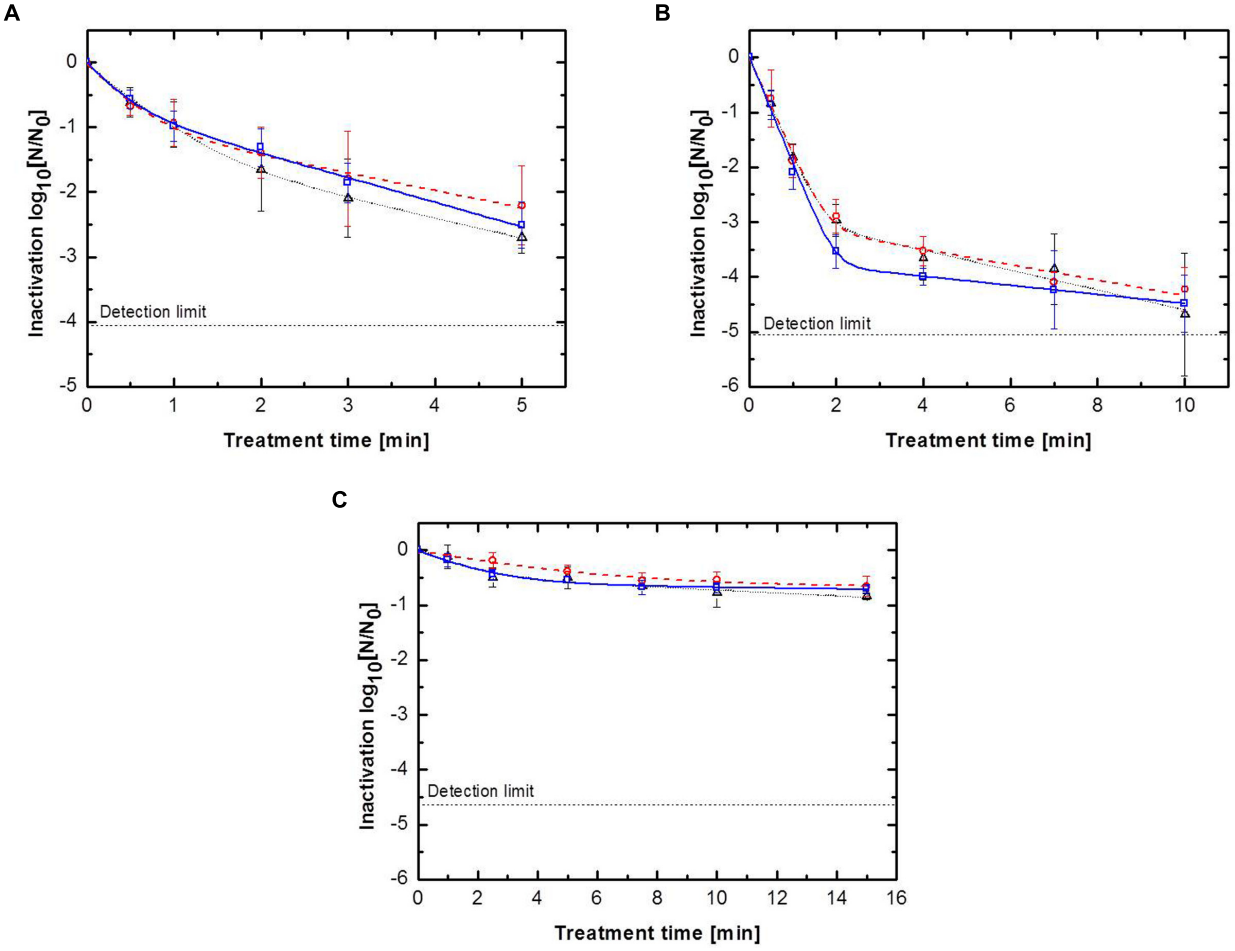
**Inactivation kinetics for *Bacillus subtilis* endospores for (△) pure argon, (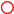) argon + 0.135% vol. oxygen, (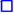) argon + 0.135% vol. oxygen + 0.2% vol. nitrogen inoculated on: **(A)** glass petri-dishes, **(B)** glass beads and **(C)** peppercorns, with biphasic fit**.

To ensure comparable results between the three surfaces, all samples were inoculated with equal endospore densities of *B. subtilis* endospores. The obtained inactivation data were modeled using a biphasic inactivation model, which adequately described the inactivation behavior (**Table 2**). The resulting inactivation kinetics are shown in **Figure 2**. All inactivation kinetics showed an accelerated initial followed by a retarded inactivation for longer plasma exposure times, which can be also seen by inactivation rate constants (k). In general the k_1_ (first inactivation phase) were higher than the k_2_ (second inactivation phase) values. For each treated surface, the achieved maximum inactivation levels were relatively close together independent of the used feed gas composition. Nevertheless, the use of pure argon as feed gas for all three different surfaces resulted in the highest inactivation level, whereas the lowest inactivation was achieved by CAPP running with argon + 0.135% vol. oxygen in all cases.

**Table 2:** Statistical parameters and the corresponding standard error (in brackets) of the biphasic model obtained from GInaFit.

	Pure argon				Argon + 0.135% vol. oxygen				Argon + 0.135% vol. oxygen + 0.2% vol. nitrogen			
	Adj. *R*^2^	φ [-]	k_1_ [min^-1^]	k_2_ [min^-1^]	Adj. *R*^2^	φ [-]	k_1_ [min^-1^]	k_2_ [min^-1^]	Adj. *R*^2^	φ [-]	k_1_ [min^-1^]	k_2_ [min^-1^]
Glass petri-dishes	1.00	0.93 (0.03)	2.54 (0.34)	0.71 (0.11)	0.98	0.86 (0.07)	3.28 (0.94)	0.62 (0.12)	0.99	0.77 (0.08)	3.85 (1.24)	0.87 (0.09)
Glass beads	0.99	1.00 (0.00)	4.15 (0.51)	0.42 (0.07)	0.99	1.00 (0.00)	4.04 (0.55)	0.32 (0.09)	1.00	1.00 (0.00)	4.48 (0.29)	0.19 (0.06)
Peppercorns	0.93	0.68 (0.12)	0.82 (0.43)	0.06 (0.03	0.96	0.76 (0.27)	0.29 (0.16)	0.01 (0.07)	0.98	0.76 (0.04)	0.64 (0.12)	0.02 (0.02)

In contrast to the feed gas composition, the treated surface had a tremendous impact on the inactivation efficiency of the CAPP plasma treatment. On the glass petri-dishes, the use of pure argon as feed gas for 5 min inactivated (**Figure 2A**) 2.7 log_10_ *B. subtilis* endospores, whereas the use of the second and third gas composition resulted in an inactivation of 2.2 and 2.5 log_10_, respectively. In contrast, the CAPP treatment of glass beads resulted in significantly higher maximum inactivation levels of about 2 log_10_ (**Figure 2B**). CAPP generated by pure argon achieved an inactivation after 10 min treatment of 4.7 log_10_, followed by argon + 0.135% vol. oxygen + 0.2% vol. nitrogen with 4.5 log_10_ and argon + 0.135% vol. oxygen with 4.2 log_10_. The higher endospore inactivation after the CAPP treatment of glass beads cannot only be explained with the longer treatment time compared to the glass petri-dishes, because the inactivation obtained after a 4 min treatment were already higher than the maximum inactivation of the glass petri-dishes, i.e., 3.7, 3.5, and 4.0 log_10_ for the three feed gas compositions. The CAPP treatment of peppercorns resulted for all three used gas compositions in an inactivation less than 1.0 log_10_ after 15 min.

### Endospore DNA Damage caused by Different Feed Gas Compositions

**FIGURE 3:**
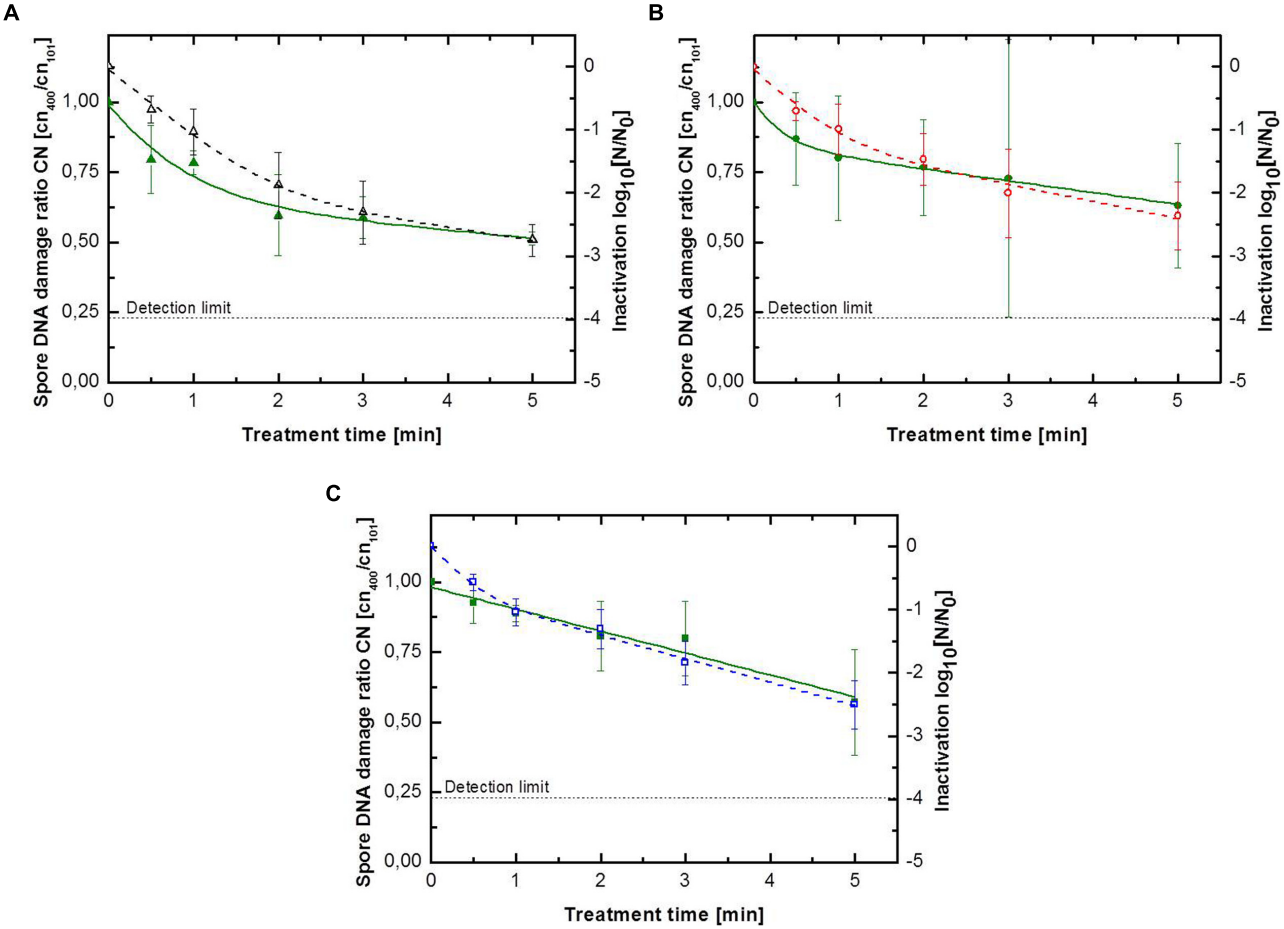
**Kinetics for *B. subtilis* endospores inoculated on glass petri-dishes for **(A)** pure argon with (△) inactivation and (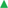)endospore DNA damage ratio, **(B)** argon + 0.135% vol. oxygen with (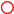) inactivation and (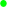)endospore DNA damage ratio, **(C)** argon + 0.135% vol. oxygen + 0.2% vol. nitrogen with (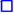) inactivation and (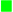) endospore DNA damage ratio.** Solid lines represent the biphasic fit for the DNA damage and the dashed lines for endospore inactivation.

**FIGURE 4:**
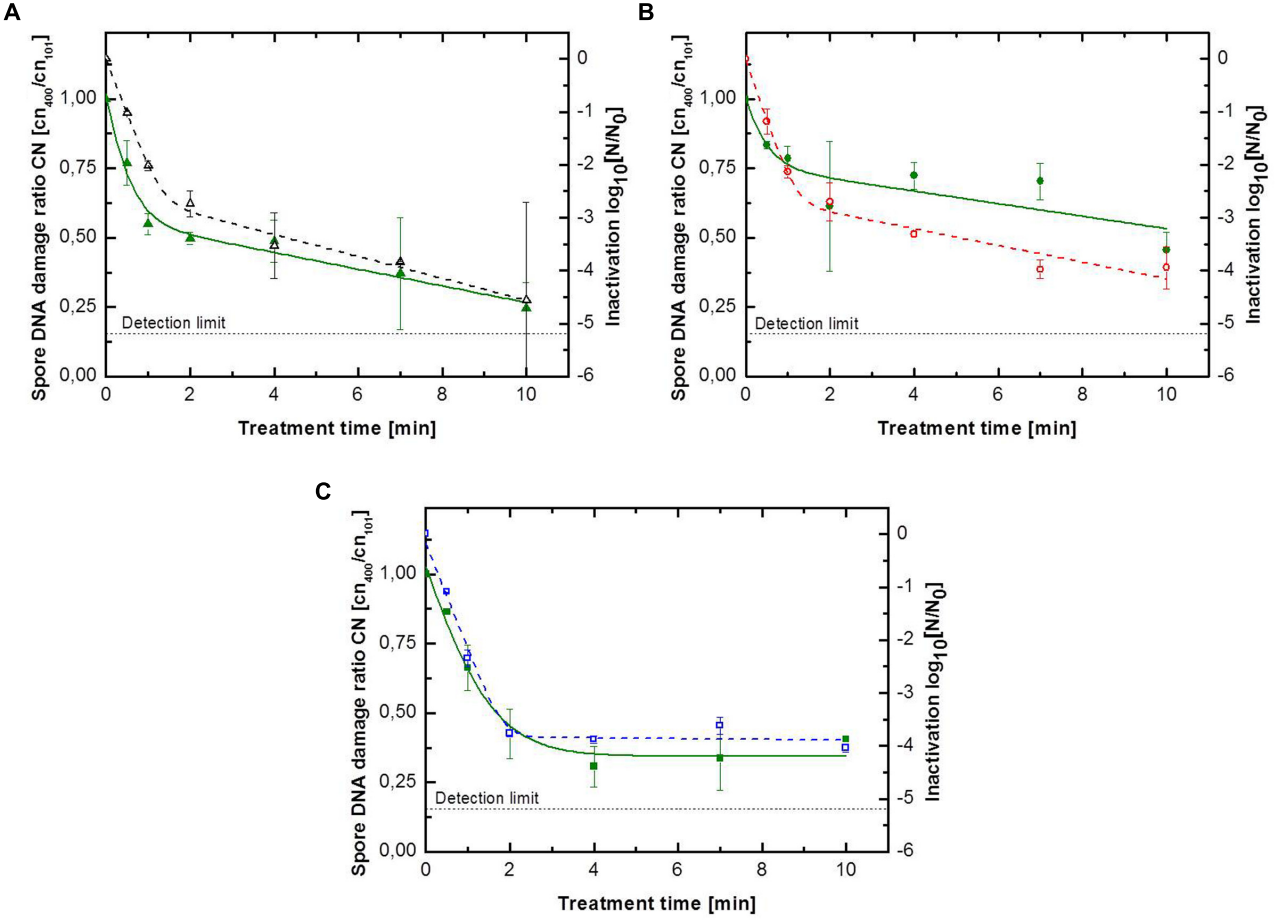
**Kinetics for *B. subtilis* endospores inoculated on glass beads for **(A)** pure argon with (△) inactivation and (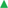)endospore DNA damage ratio, **(B)** argon + 0.135% vol. oxygen with (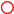) inactivation and (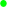)endospore DNA damage ratio, **(C)** argon 0.135% vol. oxygen + 0.2% vol. nitrogen with (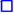) inactivation and (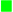) endospore DNA damage ratio.** Solid lines represent the biphasic fit for the DNA damage and the dashed lines for endospore inactivation.

In case of CAPP treated peppercorns no assessment of DNA damage was conducted. Peppercorns are often highly spoiled with microorganisms. Even though they can be sterilized, the DNA material of the native microbial load is still present on the peppercorns surface and would falsify the results. For a better comparison between the inactivation and DNA damage ratio, only inactivation data of samples, which were also used for the analyzing of the DNA damage, were considered for the depiction of the inactivation behavior. Thus the inactivation kinetics shown in **Figures 3** and **4** may differ slightly from those shown in **Figure 2**. The DNA damage ratio values were also modeled using the biphasic equation (Cerf, 1977) to investigate if the damage of the *B. subtilis* DNA during the CAPP showed a similar course as the corresponding inactivation kinetics. Furthermore, the point of inflection (PI) of the biphasic kinetics (inactivation and DNA damage) was calculated, which describe the transition between the first and the second phase and can be calculated as the point of intersection between the two linear phases.

The results for the DNA damage of *B. subtilis* endospores on glass petri-dishes are depicted in **Table 3** and **Figure 3**. The maximum DNA damage ratio correlates with the corresponding maximum inactivation level. Comparing to the inactivation kinetics not all DNA damage kinetics could be adequately described using the biphasic model, with an accelerated initial DNA damage followed by a retarded damage of DNA. The use of pure argon resulted in the highest inactivation of 2.8 log_10_ and also in the highest DNA damage with a ratio of 0.51. The shift from the accelerated first inactivation phase to the second one was at 1.9 min, whereas the shift between these phases was already at 0.1 min for DNA damage kinetic. Plasma running with argon + 0.135% vol. oxygen + 0.2% vol. nitrogen achieved after 5 min treatment an inactivation and DNA damage ratio value of 2.5 log_10_ and 0.57, respectively. The DNA damage showed a linear behavior, because k_1_ and k_2_ had the same value. The shift to the second inactivation phase was after 0.4 min. Argon + 0.135% vol. oxygen as a feed gas composition inactivated up to 2.4 log_10_ endospores and the resulting DNA damage ratio was the highest with 0.63. For DNA damage kinetic no point of inflection could be calculated, since φ was smaller than 0.5. The shift between the two inactivation phases was at about 1.0 min.

**Table 3:** Statistical parameters and the corresponding standard error (in brackets) of biphasic model obtained regarding the inactivation and DNA damage of *B. subtilis* endospores inoculated on glass petri-dishes.

	Pure argon					Argon + 0.135% vol. oxygen					Argon + 0.135% vol. oxygen + 0.2% vol. nitrogen				
	Adj. *R*^2^	φ [-]	k_1_ [min^-1^]	k_2_ [min^-1^]	PI [min]	Adj. *R*^2^	φ [-]	k_1_ [min^-1^]	k_2_ [min^-1^]	PI [min]	Adj. *R*^2^	φ [-]	k_1_ [min^-1^]	k_2_ [min^-1^]	PI [min]
Inactivation kinetic	0.99	0.98 (0.01)	2.52 (0.32)	0.48 (0.16)	1.9	0.97	0.90 (0.07)	2.92 (0.99)	0.62 (0.19)	1.0	0.99	0.79 (0.09)	4.02 (1.41)	0.85 (0.10)	0.4
DNA damage ratio kinetic	0.92	0.54 (0.16)	1.46 (0.92)	0.06 (0.09)	0.1	0.99	0.30 (0.04)	3.18 (1.05)	0.10 (0.01)	–	0.91	0.97 (8.0E+14)	0.18 (–)	0.18 (–)	–

As above mentioned, the inactivation efficiency of CAPP for the treatment of *B. subtilis* endospores on glass beads was significant higher than on the glass petri-dishes. The maximum DNA damage was also significant higher, indicated by ratio values between 0.46 and 0.25 (**Figure 4**). All DNA damage kinetics could be adequately described using the biphasic model (**Table 4**). CAPP generated with pure argon achieved again the highest inactivation level after 10 min with 4.6 log_10_, followed by the third and second gas composition with 4.0 and 3.9 log_10_ (**Figure 4**), respectively. The points of inflection for the biphasic inactivation kinetics of pure argon and argon + 0.135% vol. oxygen were relatively close together at 1.2 and 1.3 min. The accelerated first inactivation phase of argon + 0.135% vol. oxygen + 0.2% vol. nitrogen lasted until 1.9 min. The maximum values for the DNA damage showed the same trend as the maximum inactivation values. Pure argon had a ratio value of 0.25. The ones for the other two feed gas composition were considerably higher, 0.46 for argon + 0.135% vol. oxygen and 0.41 for argon + 0.135% vol. oxygen + 0.2% vol. nitrogen. No point of inflection could be calculated for the use of argon + 0.135% vol. oxygen, because φ was again smaller than 0.5. For pure argon, the shift of the two phases was already at 0.2 min, whereas the shift for the third gas composition was at 1.0 min.

**Table 4:** Statistical parameters and the corresponding standard error in brackets of biphasic model obtained regarding the inactivation and DNA damage of *B. subtilis* endospores inoculated on glass beads.

	Pure argon					Argon + 0.135% vol. oxygen					Argon + 0.135% vol. oxygen + 0.2% vol. nitrogen				
	Adj. *R*^2^	φ [-]	k_1_ [min^-1^]	k_2_ min^-1^]	PI [min]	Adj. *R*^2^	φ [-]	k_1_ [min^-1^]	k_2_ [min^-1^]	PI [min]	Adj. *R*^2^	φ [-]	k_1_ [min^-1^]	k_2_ [min^-1^]	PI [min]
Inactivation kinetic	0.99	1.00 (0.00)	4.81 (0.59)	0.48 (0.06)	1.3	0.97	1.00 (0.00)	5.20 (0.95)	0.36 (0.09)	1.2	0.98	1.00 (0.00)	4.59 (0.69)	0.02 (0.11)	1.9
DNA damage ratio kinetic	0.97	0.64 (0.05)	2.55 (0.73)	0.07 (0.02)	0.2	0.65	0.43 (0.19	2.43 (3.08)	0.05 (0.04)	–	0.96	0.79 (0.05)	1.29 (0.32)	0.00 (0.03)	1.0

## Discussion

To study the inactivation mechanisms of CAPP on endospores, *B. subtilis* endospores inoculated on different surfaces were plasma treated using three different feed gas compositions. CAPP treatment is usually described as a non-thermal process, however, during the experiment in this study peak temperatures up to 90°C were reached (**Table 1**). However, a strong thermal inactivation effect on the *B. subtilis* endospores during the CAPP treatment could not be detected in this study. The three different used gas compositions were characterized by their different emission spectra. Plasma generated by pure argon showed less emission in the UV range and of atomic oxygen than the other used feed gas compositions. The use of argon + 0.135% vol. oxygen leads to a strong emission of atomic oxygen; however, the emission in the UV range was also negligible. Plasma running with argon + 0.135% vol. oxygen + 0.2% vol. nitrogen showed a significant emission in the UV-C range, furthermore it emitted also an significant amount of reactive oxygen and nitrogen species (RNS). The emission of OH radicals reached for all three gas compositions the same range. Reineke et al. (2015) showed similar results for the emission intensities of the three used gas composition. They reported a fourfold higher UV emission by plasma running with argon + 0.135% vol. oxygen + 0.2% vol. nitrogen compared to the use of pure argon. Furthermore they also showed that the admixture of oxygen leads to an enhanced atomic oxygen emission. Regarding their different spectral intensities (**Figure 1**), different inactivation efficiencies could be expected and also different inactivation mechanisms.

In general the inactivation of endospores by CAPP treatment can be attributed to three different main mechanisms: (1) DNA damage due to UV photons, (2) intrinsic photodesorption, and (3) etching of organic molecules (Moisan et al., 2001). In case of plasma generated with argon + 0.135% vol. oxygen, which emitted the highest amount of ROS, the inactivation process should be dominated by the oxidation potential of the different ROS, namely OH radicals and atomic oxygen. In comparison, using plasma generated by argon + 0.135% vol. oxygen + 0.2% vol. nitrogen the inactivation process should be dominated by the damage of endospores DNA. A factor, which could contribute to the inactivation process are VUV photons, which can effectively inactivate *B. subtilis* endospores (Munakata et al., 1991). Argon driven plasma jets are well known to emit a certain amount of VUV light, dominated by argon excimer Ar^∗^_2_ with an intensity maximum at λ = 126 nm (Brandenburg et al., 2009; Ehlbeck et al., 2011). Brandenburg et al. (2009) characterized the VUV emission of an identically constructed argon driven plasma jet and showed that the absolute radiance in the VUV range (115–200 nm) did not change substantially up to a distance of 10 mm to the nozzle outlet and the irradiance at that distance can be estimated with about 2 Mw cm^-2^. However, the emission of VUV photons could not be measured with the spectrometer used in this study.

The results depicted in **Figure 2** indicated that the inactivation efficiency is almost independent of the used feed gas composition. On all three CAPP treated surfaces the maximum achieved inactivation of the different gas compositions was relatively similar. In contrast, the treated surface had a significant impact on the inactivation efficiency of the CAPP treatment. All different surfaces were inoculated with a similar endospore density of about 4^∗^10^6^ endospores cm^-2^ to ensure comparable results. Nevertheless, the distribution of the endospores on the surface can be different and can influence the inactivation efficiency of the plasma treatment. The significant lower inactivation of endospores on peppercorns can be explained by the structured and uneven surface. The surface of peppercorns is characterized by cracks, grooves, and pits (Hertwig et al., 2015b) and cause probably shadow effects, for the emitted (V)UV photons, reactive species and charged particles, which reduce the efficiency of the CAPP treatment.

As described above, the higher endospore inactivation for the treatment of glass beads compared to the maximum achieved inactivation on glass petri-dishes cannot only be explained with the longer treatment time. The distribution of the inoculated *B. subtilis* endospores is presumably more homogenous on glass beads than on glass petri-dishes. The endospore inoculation on the petri-dishes causes probably the formation of agglomerates on the outer edge of the endospore suspension drop. Since the penetration depth of the (V)UV photons and reactive species is restricted to a few nm, the CAPP treatment would only effect the top layer of aggregated endospores (Hertwig et al., 2015b). Noticeable is that plasma generated by pure argon, which had the lowest emission of UV photons and other reactive species, achieved on all three treated surfaces the highest inactivation. The feed gas composition argon + 0.135% vol. oxygen + 0.2% vol. nitrogen, which emitted the highest amount of UV-C photons and considerable amounts of ROS and RNS, showed on all surfaces the second highest inactivation.

Reineke et al. (2015) reported similar results for the inactivation of *B. subtilis* endospores on glass petri-dishes and showed that the use of pure argon and argon + 0.135% vol. oxygen + 0.2% vol. nitrogen for the generation of plasma resulted in a similar inactivation efficiency after a treatment of 5 min. All inactivation kinetics showed a biphasic behavior, with an accelerated first inactivation phase and a slower second one, k_1_ values were always higher than the k_2_ values (**Table 2**). The biphasic inactivation curves pointed toward different inactivation mechanisms which may be involved during the treatment. Whereas the fast inactivation during the first phase can be attributed to the emitted (V)UV photons, which cause DNA damage, the slower inactivation during the second phase is presumably caused by a combination of DNA damage, photodesorption and etching (Knorr et al., 2011).

Reineke et al. (2015) treated UV-sensitive *B. subtilis* endospore mutant strains, FB122 and PS578, using plasmas with different UV-emission intensities and showed the significant impact of UV photons on the first inactivation phase. The strain FB122 is unable to synthesize DPA during the sporulation; whereas the strain PS578 lacks the genes encoding the spore’s two major SASPs. Both DPA and SASPs contribute to the UV resistance of *B. subtilis* endospores (Reineke et al., 2015). The different weighting between the involved inactivation mechanisms can be seen in the different shift between the two inactivation phases (**Tables 3** and **4**). To further investigate the inactivation mechanisms, a qPCR based ratio detection system (Bauer et al., 2004; Roth et al., 2010) was established to monitor the damage of endospore’s DNA during the CAPP treatment. DNA is the primary target of emitted (V)UV photons during the CAPP treatment. ROS, namely atomic oxygen and OH radicals, are well known for their oxidation potential and can react with almost all cell components (Surowsky et al., 2014). Whereas OH radicals have the highest oxidation potential of all ROS, they are able to oxidize unsaturated fatty acids, proteins and to initiate DNA damage (Laroussi, 2002; Surowsky et al., 2014). However, the saturation of *B. subtilis* endospores DNA with α/β-type small acid soluble proteins (SASP) protects the DNA against damage caused by OH radicals (Setlow and Setlow, 1993). Hence, the measured DNA damage can be attributed to the emitted (V)UV photons. The DNA damage ratio values showed the same dependence on the used feed gas composition like the maximum achieved inactivation. The fact that plasma running with pure argon obtained on both surfaces the highest DNA damage showed that VUV photons can play a significant role in the inactivation of endospores at atmospheric pressure. All DNA damage kinetics, except the one of argon + 0.135% vol. oxygen + 0.2% vol. nitrogen on glass beads, showed a continuous increase of DNA damage during the entire inactivation process. The DNA damage kinetic for the treatment on glass beads using plasma running with argon + 0.135% vol. oxygen + 0.2% vol. nitrogen merge into a tailing after 3 min (**Figure 4C**). However, the corresponding inactivation curve showed the same tailing, this behavior support the finding, that the damage of DNA due to (V)UV photons is one of the main inactivation mechanisms.

The point of inflection between the two inactivation phases was achieved always significantly earlier for the DNA damage kinetics than for the corresponding inactivation kinetics (**Tables 3** and **4**). The accelerated first inactivation phase is often attributed to the damage of DNA by (V)UV photons (Moisan et al., 2002), however, the different shift between the two phases for the inactivation and DNA damage showed that the (V)UV based inactivation is presumably not the only mechanism contributing to the first fast inactivation phase. The linear behavior for the DNA damage kinetic of argon + 0.135% vol. oxygen + 0.2% vol. nitrogen (**Figure 3C**) can be explained by the high amount of emitted ROS and RNS. High amounts of reactive species are necessary for the decomposition of endospores due to etching, which lead to exposure of former covered endospores in, e.g., agglomerates and consequently to a further DNA damage. The early shift in the inactivation kinetic at 0.4 min may indicate the point, where the inactivation based on UV photons change to an inactivation process based on UV photons and supported by etching and photodesorption. For both DNA damage kinetics of argon + 0.135% vol. oxygen (glass beads and glass petri-dishes) the shift between the two phases could not be calculated, because both φ were under 0.5 and the treatment resulted in the highest DNA damage ratio value for both surfaces. Plasma generated with argon + 0.135% vol. oxygen emitted the highest amount of ROS, furthermore the admixture of air to argon decreases the VUV emission due to an increase of atomic oxygen emission (Brandenburg et al., 2009). Considering this, the inactivation process seems to be dominated by the action of ROS.

## Conclusion

It can be stated that (V)UV photons emitted by CAPP play presumably a considerable role during the inactivation process of *B. subtilis* endospores, if the (V)UV intensity is high enough. Furthermore, the structure of the plasma treated surface as well as the distribution of the endospores on it affects the inactivation efficiency of CAPP treatment.

## Conflict of Interest Statement

The authors declare that the research was conducted in the absence of any commercial or financial relationships that could be construed as a potential conflict of interest.
